# Effect of substrate stiffness on friction in collective cell migration

**DOI:** 10.1038/s41598-022-06504-0

**Published:** 2022-02-15

**Authors:** Kelly Vazquez, Aashrith Saraswathibhatla, Jacob Notbohm

**Affiliations:** 1 Department of Engineering Physics, University of Wisconsin-Madison, Madison, WI USA; 2 Department of Mechanical Engineering, University of Wisconsin-Madison, Madison, WI USA

**Keywords:** Biomedical engineering, Biological physics, Collective cell migration

## Abstract

In collective cell migration, the motion results from forces produced by each cell and transmitted to the neighboring cells and to the substrate. Because inertia is negligible and the migration occurs over long time scales, the cell layer exhibits viscous behavior, where force and motion are connected by an apparent friction that results from the breaking and forming of adhesive bonds at the cell–cell and cell–substrate interfaces. Most theoretical models for collective migration include an apparent friction to connect force and motion, with many models making predictions that depend on the ratio of cell–cell and cell–substrate friction. However, little is known about factors that affect friction, leaving predictions of many theoretical models untested. Here, we considered how substrate stiffness and the number of adhesions affected friction at the cell–substrate interface. The experimental data were interpreted through prior theoretical models, which led to the same conclusion, that increased substrate stiffness increased the number of cell–substrate adhesions and caused increased cell–substrate friction. In turn, the friction affected the collective migration by altering the curvature at the edge of the cell layer. By revealing underlying factors affecting friction and demonstrating how friction perturbs the collective migration, this work provides experimental evidence supporting prior theoretical models and motivates the study of other ways to alter the collective migration by changing friction.

## Introduction

Collective cell migration occurs in wound healing^1–3^, cancer invasion^4–6^, and development^7,8^. The motion results from forces produced within each cell that are transmitted to the cell–cell and cell–substrate interfaces. To produce motion, those forces balance not with inertia, which is negligible, but rather with a frictional force that resists motion^9–19^. The underlying source of friction in a cell layer is that cell motion requires breaking of current adhesions and forming of new adhesions; because adhesions have a characteristic lifetime, some adhesions may resist breaking, thus producing a force that resists motion. Specific details on the form of the equation relating frictional force and motion are not yet clear, but many models use a viscous-type friction, with good agreement between model predictions and experiments^10–16^. Elastic contributions to the forces are negligible in this case, as the migration occurs on long time scales^11^. The frictional force can exist either at the cell–cell junctions, in which case we refer to it as tissue viscosity, or the cell–substrate interface, in which case we refer to it as cell–substrate friction. Viscosity and friction form the fundamental connection between force and motion in collective cell migration, and there exists circumstantial evidence suggesting that viscosity and friction may have important effects on the overall patterns of migration. For example, reducing cell–cell adhesions tends to reduce the distance over which each cell’s motion is correlated with that of its neighbors^20,21^. Although the biophysical mechanism by which cell–cell adhesions affect the migration is not yet clear, a possible explanation is that the reduced cell–cell adhesions reduced the viscosity in the cell layer. This explanation has not been tested, however, because it remains unknown whether the reduced adhesions affected viscosity and friction. This example is representative of the need for fundamental understanding of how different perturbations to the cell collective affect viscosity and friction.

Initial insight comes from the seminal theory of Schallamach^22^, which considered friction of rubber sliding over a surface as resulting from the forces at individual bonds. According to the theory, the friction force is equal to *Nkvτ*, where *N* is the number of bonds, *k* is the stiffness, *v* is the sliding velocity, and *τ* is the average bond lifetime. Due to the dependence on velocity, this relationship is inherently viscous-like, connecting force and motion. The equation force = *Nkvτ*, can be applied to cell migration as well^11,12^, though with the added complication that each variable in the equation can be actively controlled by the cell. For example, the stiffness *k* represents the combined stiffness of the cell, bond, and substrate, and cells can adapt their stiffness to match that of the substrate^23^. Additionally, the active response of the cell creates complicated relationships between different variables in this equation, with evidence for biphasic relationships between force and actin flow velocity^24–26^ or between force and substrate stiffness^27,28^. Further insight comes from estimates of the ratio between viscosity and friction. Theoretical models for collective cell migration^11–13,16–19,29,30^ define viscosity *η* as the ratio of stress and velocity gradient (i.e., the difference in velocity) at the cell–cell interface with units of (force)(time)/(length)^2^ and friction *ξ* as the ratio of the cell–substrate force density (i.e., force per cell volume) and cell velocity with units of (force)(time)/(length)^4^. Hence, the resulting number $\lambda = \sqrt {\eta /\xi }$ has units of length and describes the distance over which the motion of one cell affects the motion of another cell.

The magnitude of *λ* is still unclear, because it is not possible to measure viscosity or friction directly. The inherent complication is that tractions at the cell–substrate interface and stresses within the cell monolayer result from a combination of passive viscous terms and active forces produced by each cell. In particular, traction is a sum of friction and active propulsive forces; similarly, stresses are a sum of an effective viscosity and the active contraction of the cell^30^. Because friction and viscosity cannot be decoupled from the active contributions of the cell, they cannot be measured directly, meaning it is not yet possible to measure *λ* directly. Some studies have proposed that friction dominates over viscosity, in which case *λ* would be small, ranging from a single cell to a few cells in size^13,17^. Other studies found the ratio of viscosity to friction to be large, with *λ* on the order of hundreds of microns or larger^12,19,31^. These conflicting experimental observations are likely caused in part by different experimental conditions. For example, with increasing time in culture, cell adhesions may evolve such that viscosity becomes larger than friction^14^. There is also evidence that the ratio of viscosity to friction increases over time in *Drosophila* epithelium^16^. Other experimental conditions affecting the balance between viscosity and friction remain unknown, which highlights the need for studies to systematically perturb viscosity or friction and to quantify the resulting effect on the collective migration.

In this study, we conducted experiments to perturb the friction in cell monolayers. Following the equation that friction force is equal to *Nkvτ*, we hypothesized that increasing the substrate stiffness would increase the friction. The results were interpreted by comparison to two different theoretical models, the first by Garcia et al. predicting a biphasic relationship between velocity correlation length and speed, depending on the balance of viscosity and friction^14^, and the second by Alert et al. predicting that length scale *λ* is proportional to the size of a protrusion at the edge of the cell layer^18^.

## Results

### Increased substrate stiffness increases cell–substrate adhesions

Starting with the equation force = *Nkvτ,* we hypothesized that friction may be affected by substrate stiffness through two mechanisms. First, variable *k* represents the combined stiffness of the cell, bonds, and substrate^22^, and given that cells can adapt their stiffness to match that of the substrate^23^, it is feasible that all are of the same order of magnitude, suggesting that increasing substrate stiffness would directly increase variable *k*, thereby increasing the friction force. Secondly, cells respond actively to substrate stiffness by changing the number and size of focal adhesions^32^, meaning that increasing substrate stiffness is likely to increase the number of adhesive bonds *N*, which would also increase the friction force. Consistent with this notion are prior studies, which suggested that greater focal adhesion area is associated with greater friction^14,33^.

**Figure 1:**
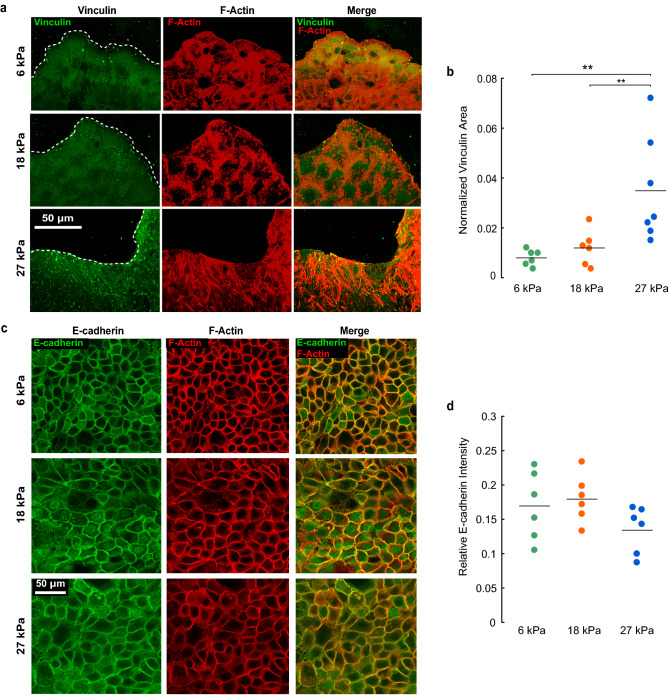
Effect of substrate stiffness on cell–substrate and cell–cell adhesions. (**a**) Confocal images of HaCaTs stained for vinculin (green) and actin (red) on substrates having moduli of 6, 18, and 27 kPa. (**b**) Effect of substrate stiffness on normalized vinculin area. (**c**) Confocal images of HaCaTs stained for E-cadherin (green) and actin (red) for varied substrate stiffness. (**d**) Relative E-cadherin intensity (difference between junctional and cytoplasmic intensities divided by the sum) for varied substrate stiffness. No statistical effect of substrate stiffness on relative intensity was present. For panels (**b**,**d**), each data point represents an average over a statistically independent field of view. Horizontal lines indicate means.

To investigate the relationship between substrate stiffness and number of bonds *N*, we fabricated substrates with Young’s moduli of 6, 18, and 27 kPa. We seeded human keratinocyte cells (HaCaTs) onto the substrates next to barriers with a straight edge. The barriers were made of polydimethylsiloxane (PDMS) embedded with iron filings and were held in place with magnets beneath the cell culture dish^34^ and removed by placing another magnet over the top of the dish (Supplemental Fig. S1). Release of the barriers created free space, modeling a wound, which induced collective cell migration^35,36^. After allowing the cells to migrate for 24 h, we fixed and stained for actin and vinculin. The images showed that cells on the 6 kPa and 18 kPa substrates had few focal adhesions, with the largest adhesions appearing at the edge of the cell monolayer (Fig. 1a). By contrast, cells on 27 kPa substrates had clear and elongated focal adhesions that were present both at the monolayer edge and in the bulk (Fig. 1a). We binarized the images of vinculin (Supplemental Fig. S2), which enabled us to quantify a normalized vinculin area, defined as the ratio of the area containing vinculin to the total area covered by the cell layer. With increased substrate stiffness, the normalized vinculin area increased by 4.4-fold and 3.1-fold for cells on 27 kPa substrates compared to 6 kPa and 18 kPa substrates, respectively (Fig. 1b). The increased vinculin area implies a greater number of cell–substrate bonds *N*, consistent with our expectation.

Considering that substrate stiffness could also affect cell–cell adhesions, we also quantified the presence of E-cadherin at the cell–cell junctions by fluorescently staining for actin and cadherin in cells on substrates of different stiffness. Cells on substrates of all three stiffness had E-cadherin localized at the cell–cell junctions (Fig. 1c). E-cadherin images were analyzed by computing the fluorescent intensity of cadherin at the cell–cell junctions and inside the cytoplasm of each cell. Quantification of relative E-cadherin fluorescent intensity (difference between junctional and cytoplasmic intensities divided by the sum) showed no statistical difference, suggesting no effect of substrate stiffness on cell–cell adhesions (Fig. 1d).

In summary, there are two possible mechanisms by which substrate stiffness affects friction. The first is the direct effect on variable *k* in the expression *Nkvτ*. The second is indirect and results from the active cell response to increased substrate stiffness by creating more and larger focal adhesions, thereby increasing the variable *N*. Both mechanisms lead to the same prediction, that increased substrate stiffness would increase the friction.

### Effect of substrate stiffness on root-mean-square velocity and velocity correlation length

With the effects of substrate stiffness on the cell–substrate and cell–cell adhesions established, we next designed an experiment to test the main hypothesis in this study, that increasing substrate stiffness increases the friction. Since it is not possible to measure friction directly, we designed experiments based on the results of Garcia et al.^14^, who studied the balance between viscosity and friction. Using a combination of experiments and theory, Garcia et al. established that viscosity and friction jointly affect the relationship between the root-mean-square (RMS) velocity and the velocity correlation length, which describes the characteristic size of a collectively migrating pack of cells within the confluent cell layer. In their experiments, Garcia et al. found that a plot of velocity correlation length against RMS velocity formed a distinct bell-shaped curve. To investigate the cause of this relationship, they formulated three different models of increasing complexity. The simplest model considered groups of cells moving together in the confluent layer in two limits. The first limit corresponded to viscosity being far larger than friction, in which case the RMS velocity increased with increasing correlation length, meaning the slope of a curve of velocity correlation length against RMS velocity was positive. The second limit corresponded to friction being far larger than viscosity, for which a plot of velocity correlation length against RMS velocity had a slope that was negative. The second and third models, which studied in closer detail the cell adhesions and the overall motion of the collective, connected these two limits, creating the bell-shaped curve that matched the experimental data. Hence, a plot of velocity correlation length against RMS velocity offers a means to determine the relative balance between viscosity and friction: on the left side of the curve, the slope is positive, which indicates viscosity is dominant; on the right side, the slope is negative, indicating friction is dominant. In between, where the slope is flat, viscosity and friction have similar magnitudes.

**Figure 2:**
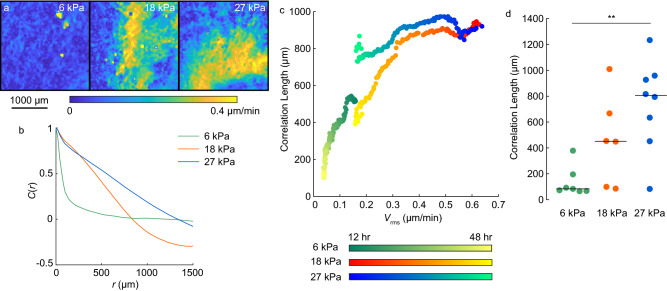
Effect of substrate stiffness and time in culture on RMS velocity and velocity correlation length. (**a**) Representative color maps of speed of cells on substrates of moduli 6, 18, and 27 kPa at *t* = 45 h after the cells reached confluence. (**b**) Autocorrelation of cell velocity *C*(*r*) plotted as a function of separation distance *r* at *t* = 45 h for a representative cell layer on each substrate stiffness. (**c**) Velocity correlation length plotted against RMS velocity. The brightness indicates time after confluence and the color family indicates substrate stiffness as indicated in the legend. Each data point represents the average of at least 6 statistically independent samples on each substrate stiffness. (**d**) Velocity correlation length for each substrate stiffness. Data shown represent averages over time points collected in the range *t* = 42–48 h after confluence. Each dot represents an independent measurement; horizontal lines indicate means.

To assess how the balance between friction and viscosity depended on substrate stiffness, we implemented the experiments of Garcia et al., seeding cells in large confluent layers on substrates of different modulus (6, 18, and 27 kPa), imaging the center of them for 48 h, and using image correlation to quantify the cell velocities. On the softest substrate (6 kPa), cells generally moved slowly with large fluctuations in cell speed over space. With increasing stiffness, speeds increased, and spatial fluctuations decreased (Fig. 2a). To quantify the spatial fluctuations, we computed a spatial autocorrelation of the velocity (see “Methods” for details). Representative curves suggested that the autocorrelation was greater on stiffer substrates (Fig. 2b), consistent with the smaller spatial fluctuations observed in Fig. 2a. In fact, it was common in experiments of high substrate stiffness for the velocity correlation length to be so large that the field of view contained only 2–3 large groups of collectively moving cells (Supplemental Fig. S3). As these groups moved in different directions, the velocity autocorrelation became negative at large distances, approximately equal to the size of a collectively moving group of cells (Fig. 2b). From the velocity autocorrelation, we calculated the velocity correlation length, defined as the distance over which the autocorrelation decreased to a value of 0.2.

We then computed the correlation length and RMS velocity and averaged over all cell monolayers for each substrate stiffness and time point. The resulting data set had four dimensions, with correlation length vs. RMS velocity depending on both time and substrate stiffness. The data were plotted with different colors with the brightness indicating time in culture and the color family indicating the substrate stiffness (Fig. 2c). For early time points, the data from the 18 and 27 kPa substrates had a slope that was flat or, for 27 kPa, even slightly negative, indicating an approximately equal balance between viscosity and friction for those data sets. The curve corresponding to the 6 kPa substrates always had a positive slope and never become flat, indicating a greater ratio of viscosity to friction on substrates of 6 kPa compared to 18 or 27 kPa. To verify that the differences observed in Fig. 2c were statistically significant, we averaged the correlation length for each cell monolayer over time points spanning *t* = 42–48 h and plotted the results (Fig. 2d). The correlation length on the 27 kPa substrates was statistically larger than on 6 kPa substrates (Fig. 2d), which is consistent with prior reports of the effect of substrate stiffness on velocity correlation length^37^. Together, our data, interpreted through the models by Garcia et al., suggest that with increased substrate stiffness, the cell monolayers transitioned from a state wherein friction was smaller than viscosity to a state wherein friction and viscosity were of similar magnitude. Given that the change in substrate stiffness had no effect on cell–cell adhesions (Fig. 1c,d), it is likely that changes in viscosity were negligible, which would imply that the increased substrate stiffness caused an increase in friction.

### Effect of time in culture on cell–substrate and cell–cell adhesions

An interesting trend in the data in Fig. 2 was that, with increasing time, the data moved toward the origin on the plot, suggesting the ratio of viscosity to friction increased over time. This trend was observed by Garcia et al.^14^ as well, who attributed it to maturation of adhesions such that with increasing time, the effect of cell–cell adhesions dominated over that of cell–substrate adhesions. This finding is consistent with other studies that used a continuum model combined with experiments to suggest that viscosity increases over time in a cell layer^11,31^. These prior studies motivated us to verify that effects of time in culture in our experimental system were consistent with the prior literature.

**Figure 3:**
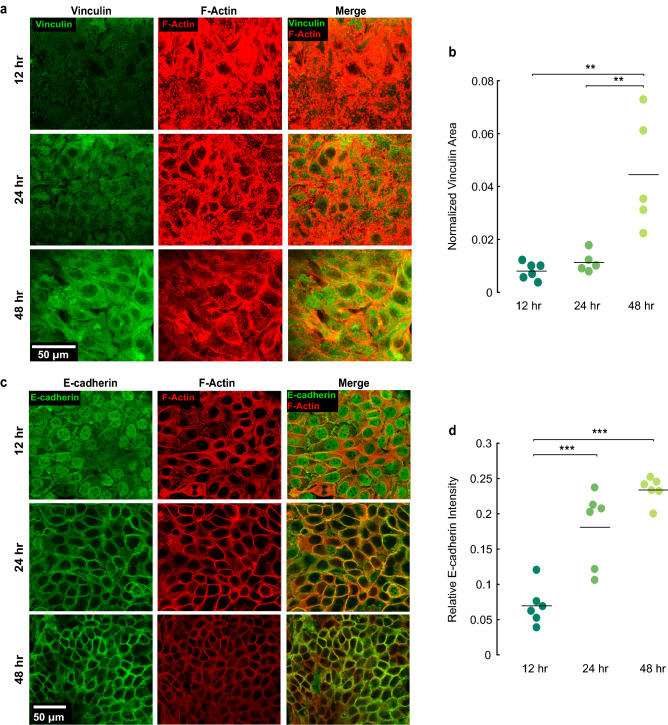
Effect of time in culture on cell–substrate and cell–cell adhesions. (**a**) Confocal images of HaCaTs stained for vinculin (green) and actin (red) after 12, 24, or 48 h in culture on substrates of Young’s moduli 6 kPa. (**b**) Effect of time in culture on normalized vinculin area. (**c**) Confocal images of HaCaTs stained for E-cadherin (green) and actin (red) for varied times in culture. (**d**) Relative E-cadherin intensity (difference between junctional and cytoplasmic intensity divided by the sum) for varied time in culture. For panels (**b**,**d**), each data point represents an average over a statistically independent field of view. Horizontal lines indicate means.

Cell–substrate focal adhesions were assessed as in Fig. 1, by staining for vinculin and quantifying the normalized vinculin area for cells seeded on substrates with Young’s moduli 6 kPa. With increasing time in culture, the focal adhesions evolved from being distinct, punctate structures to being uniformly distributed under the cell monolayer (Fig. 3a). Quantification showed that the normalized vinculin area for cells cultured for 24 h and 48 h was 1.7-fold and 5.1-fold greater than for cells cultured for 12 h (Fig. 3b). As in Fig. 1, the increase in vinculin area could indicate more focal adhesions and greater friction. However, the uniform distribution of vinculin at long times in culture led us to consider that the images of vinculin alone may not have been representative of the friction—for vinculin intensity to correspond to friction, the vinculin structures would have to be connected to actin stress fibers supporting force. Images of actin showed progressive decreases in actin at the base of the cell layer over time (Fig. 3a), suggesting reduced force at the cell–substrate interface at later times. Cell–cell adhesions were assessed by fluorescent staining for actin and cadherin in cells cultured for different times. Unlike cells cultured for a short time (12 h), cells cultured for longer culture times (24, 48 h) had clear localization of E-cadherin at the cell–cell junctions (Fig. 3c). Quantification of the relative E-cadherin intensity showed 2.6-fold and 3.4-fold increases for 24 and 48 h in culture compared to 12 h in culture, respectively (Fig. 3d). Thus, increased time in culture resulted in an increase in both cell–cell and cell–substrate adhesions. This finding is consistent with results of Garcia et al.^14^ if in our data, the viscosity increased more so than the friction, which is reasonable, considering the time-dependent reduction in force-supporting actin at the cell–substrate interface.

For additional evidence that viscosity increased more so than friction with increasing time in culture, we turned to a prior report suggesting that in cell monolayers having a large ratio of cell–cell to cell–substrate adhesion, a multicellular actin cable can form at the edge of the monolayer^33^. The actin cable affects the formation of leader cells and the rate of wound closure^38,39^. To check for an actin cable, we cultured cells for 12–72 h against flat barriers, removed the barriers, and fixed and stained for F-actin. For ≤ 24 h in culture, no actin cable was present (Supplemental Fig. S4a), nor did one appear after a subsequent 24 h of migration (Supplemental Fig. S4b). At 48 h in culture, segments of the cable were present with a complete cable forming at 72 h in culture (Supplemental Fig. S4a). For these long times in culture, the actin cable remained even for an additional 24 h of migration (Supplemental Fig. S4c). These observations of the multicellular actin cable for long times in culture give additional evidence supporting the idea that with increased time in culture, the balance of cell–cell and cell–substrate adhesions shift such that cell–cell adhesions became dominant, consistent with the findings of Garcia et al.^14^.

### Distance between protrusions at the leading edge of the cell monolayer

Having found evidence that increased substrate stiffness increased the friction, we next sought to verify this finding using a different experimental system. Given that length scale *λ*, which depends on the ratio of viscosity to friction, describes the distance over which viscous stresses propagate through the cell layer, it may seem that length *λ* would be proportional to the correlation length of velocity. However, other physical factors could also affect the velocity correlation length, including the correlation lengths of stresses within the monolayer^37,40,41^ and traction at the cell–substrate interface^42^. Therefore, we turned to predictions of another theoretical model, this one by Alert et al., that the length scale *λ* is proportional to the width of a protrusion at the leading edge of a monolayer^18^.

**Figure 4:**
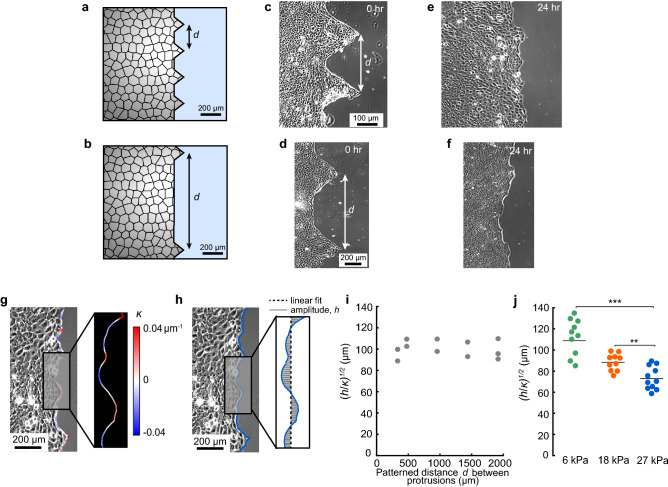
Micropatterning of cell features with varied spacing and quantification of $\sqrt {h/\kappa }$ at leading edge. (**a**,**b**) Schematic of cell layer having patterned distance, *d*, of 250 μm (**a**) and 1000 µm (**b**) between protrusions. (**c**,**d**) Representative phase contrast images of HaCaTs patterned with *d* = 250 μm (c) and *d* = 1000 µm (**d**) immediately after barrier removal. (**e**,**f**) Representative phase contrast images of HaCaTs patterned with* d* = 250 μm (**e**) and *d* = 1000 µm (**f**) at 24 h after barrier removal. (**g**) Representative phase-contrast image of leading edge of cell layer after 24 h of migration. The local curvature, κ, at the leading edge is overlaid on the phase contrast image in color. (**h**) Representative phase-contrast image of leading edge. The linear fit and the amplitude, *h*, of the leading edge are shown where the amplitude is the orthogonal distance from the linear fit to the leading edge. (**i**) The metric $\sqrt {h/\kappa }$ of the leading edge after 24 h of migration, which is predicted to be proportional to *λ*, is plotted against the initial patterned distance *d* between protrusions. (**j**) $\sqrt {h/\kappa }$ after 24 h of migration for cells seeded against a barrier with a flat edge on substrates of varied stiffness. Each data point represents an independent measurement. Horizontal lines indicate means.

We began by reasoning that if the characteristic width of a protrusion depends on viscosity and friction through variable *λ*, then the initial geometry at the leading edge of the cell layer should not affect the characteristic width of a protrusion after several hours of cell migration. Therefore, we designed an experiment to create protrusions at the edge of a cell monolayer separated by a controlled distance *d* using PDMS barriers. The barriers patterned the cell layer with pointed features of spacing *d* ranging from 250 to 2000 μm (Fig. 4a,b). HaCaTs were seeded on polyacrylamide substrates of Young’s modulus 6 kPa next to the barriers and allowed to reach confluence prior to starting the experiment. At time *t* = 0 h we removed the barriers and imaged the leading edge to verify that the cell layer was undamaged (Fig. 4c,d). After 24 h of migration, we imaged again. Comparison of the images at *t* = 0 and 24 h showed that after migration over 24 h, the spacing between protrusions at the leading edge of the cell layer did not reflect the initially patterned features (Fig. 4e,f). To quantify this observation, we used the fact that the distance between protrusions at the edge of the cell layer is equivalent to a wavelength, which is proportional to $\sqrt {h/\kappa }$, where *h* is the amplitude and *κ* is the curvature of the leading edge of the cell layer. Hence, the model of Alert et al. would predict that $\lambda = \sqrt {h/\kappa }$, meaning $\sqrt {h/\kappa }$ gives a readout for length *λ*. We therefore quantified curvature and amplitude (Fig. 4g,h) and computed $\sqrt {h/\kappa }$ in cell monolayers having different initial spacing *d*. The values of $\sqrt {h/\kappa }$ were uncorrelated to the initial distance (Fig. 4i), suggesting that $\sqrt {h/\kappa }$ was an inherent property of the cell layer. Importantly, this finding is consistent with the predictions of Alert et al., as *λ* depends on viscosity and friction but not on the initial shape of the cell layer.

We next tested whether $\sqrt {h/\kappa }$, which is a readout for *λ*, was affected by substrate stiffness. We again seeded HaCaTs next to PDMS barriers on substrates of different modulus (6, 18, and 27 kPa). Importantly, this time the barriers had flat edges, which better matched the initial conditions of the model by Alert et al.^18^. We cultured the cells for 12 h and removed the barriers, allowing the cells to migrate for 24 h. The chosen timings, culture time of 12 h and subsequent migration of 24 h, were too short for a multicellular actin cable to form (Supplemental Fig. S4), meaning the experiments were unaffected by the fact that leader cells form at breaks in the actin cable^39^. Time-lapse imaging during the 24 h of migration showed that the increased substrate stiffness affected the overall motion, causing increases in average cell persistence and directionality (Supplemental Fig. S5), consistent with a prior study that showed greater cell persistence and directionality on stiffer substrates^43^. Consistent with our prediction, the increased substrate stiffness had an effect on $\sqrt {h/\kappa }$ at the leading edge, with increasing substrate stiffness causing smaller $\sqrt {h/\kappa }$, indicating smaller *λ* (Fig. 4j). To confirm this finding, we also manually measured the distance between protrusions at the monolayer edge, observing smaller distances on substrates of greater stiffness (Supplemental Fig. S6). These data indicate that length λ decreased with increasing substrate stiffness, which, together with the finding that substrate stiffness did not affect cell–cell adhesions, leads to the conclusion that increased substrate stiffness caused increased friction.

**Figure 5:**
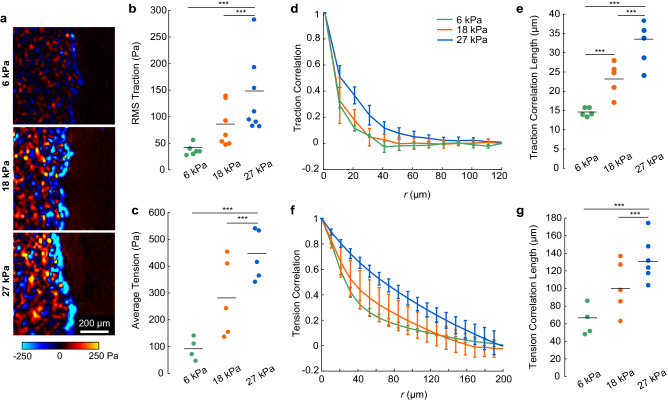
Correlation length of tractions and monolayer tension. (**a**) Representative color maps of the component of cell-to-substrate traction perpendicular to the monolayer edge for each substrate stiffness. (**b**,**c**) RMS traction (**b**) and average monolayer tension (**c**) for varied substrate stiffness. (**d**) Spatial autocorrelation of traction plotted as a function of separation distance *r* for cell layers on each substrate stiffness. (**e**) Correlation length of traction. (**f**) Autocorrelation of monolayer tension for each substrate stiffness. (**g**) Correlation length of monolayer tension. In panels d and f, lines and error bars show mean ± standard deviation over at least 4 independent samples. In panels (**b**,**c**,**e**,**g**), dots represent independent measurements, and horizontal lines indicate means.

A possible alternative explanation for the altered $\sqrt {h/\kappa }$ on substrates of different stiffness is that $\sqrt {h/\kappa }$ may have been related to a different inherent length in the cell monolayer, for example, the distance over which forces propagate in the cell layer, as observed previously^37^. To consider this possibility, we used traction force microscopy and monolayer stress microscopy to compute, respectively, the cell–substrate tractions and the tension, defined as the average of the principal stresses within the plane of the monolayer. With increased substrate stiffness, the RMS traction and average tension increased by factors of 3.8 and 4.6, respectively, from the lowest to highest substrate stiffness (Fig. 5a–c). Following methods established in our recent manuscript^42^, we also tracked cell nuclei, which enabled us to approximate the boundary of each cell with a Voronoi tessellation. From the approximated cell boundaries, we mapped the field of traction to each cell and took the vector sum of traction applied by each cell, thereby giving a net traction applied by that cell. From the vector sum, we computed an autocorrelation, which typically decayed to zero over a distance of ≈40–60 µm (≈2–3 cell widths), indicating that cells coordinate with their neighbors to apply traction in the same direction (Fig. 5d), as in our recent work^42^. From the autocorrelation of net traction, we quantified the traction correlation length, which increased by a factor of 2.3 on substrates of modulus 27 kPa compared to 6 kPa (Fig. 5e). Similarly, the correlation length of tension increased with increasing substrate stiffness (Fig. 5f,g). The increased correlation lengths with increasing substrate stiffness are consistent with prior work showing an increase in the tension correlation length with increasing substrate stiffness^37^ and are reminiscent of the effect of substrate stiffness on velocity correlation length in our data (Fig. 2d). However, these results do not explain the observation of decreased $\sqrt {h/\kappa }$ on substrates of greater stiffness (Fig. 4j), because $\sqrt {h/\kappa }$ has units of length, meaning the trends of traction and stress correlation length are opposite to those of $\sqrt {h/\kappa }$. Hence, the data are contrary to the alternative explanation, which gives supporting evidence to the idea that $\sqrt {h/\kappa }$ at the edge of the cell layer is proportional to length scale *λ*. These findings give the first experimental support for the prediction of Alert et al.^18^, that length scale *λ* affects the characteristic size of a protrusion at the edge of a cell layer.

## Discussion

Collective cell migration is sometimes described in terms of a balance between cell–cell and cell–substrate interactions^14,18,19,33,44–46^. In this manuscript, we focused on one aspect of those interactions, the resisting force that creates apparent viscosity and friction at the cell–cell and cell–substrate interfaces. Because viscosity and friction cannot be measured directly in cell monolayers, our study used experiments that were informed by theory. Motivated by the equation friction force = *Nkvτ*, we hypothesized that increasing the substrate stiffness would increase friction. In an initial set of experiments, we demonstrated that increasing substrate stiffness increased the number of cell–substrate adhesions, suggesting that increasing substrate stiffness potentially increased both *k* and *N*. We next tested our hypothesis using experiments matching the study by Garcia et al. that used RMS velocity and velocity correlation length to identify the relative balance between viscosity and friction^14^. The results indicated that increasing substrate stiffness shifted the data to a regime in which the ratio of viscosity to friction was reduced, consistent with our hypothesis of increased friction caused by increased substrate stiffness. Finally, we designed experiments to test the model by Alert et al., which shows length scale *λ* sets the distance between protrusions at the edge of the cell layer^18^. Using $\sqrt {h/\kappa }$ as a readout for *λ,* we observed *λ* to decrease with increasing substrate stiffness, also consistent with greater friction caused by increased substrate stiffness.

A central limitation of our study was that friction in the cell layer cannot be measured directly, meaning interpretation of the results was indirect. To mitigate this limitation, our study interpreted the experimental results through two prior models, built using different theoretical frameworks. The first model, by Garcia et al., was based on a combination of analytical theory for cells moving in clusters due to active cell forces and particle-based simulations^14^. The second model, by Alert et al., was based on a continuum model for an active polar fluid^18^. Although the second model has not yet been confirmed by experiments, predictions of the first model were verified by experiments performed by Garcia et al. Our experiments agree with the experiments of Garcia et al. and the predictions of both models, meaning that our data give the first experimental confirmation of predictions of the model by Alert et al. Additionally, the conclusions that came from interpreting our experimental data through the two models were the same, namely that increasing substrate stiffness increased the ratio of friction to viscosity. This information, together with our data showing no effect of substrate stiffness on E-cadherin, provides evidence that increasing substrate stiffness increased the friction between the migrating cell layer and the substrate.

Our observation of increased substrate stiffness causing decreased $\sqrt {h/\kappa }$ (Fig. 4j) may appear to conflict with a prior study that found greater substrate stiffness to increase the distance between leader cells at the edge of the monolayer^37^. However, leader cells are biologically distinct from other cells in the monolayer, in that leaders have larger lamellipodia, different protein expression including upregulation of Delta1, and, sometimes, a multicellular actin cable connecting the leader to the followers^38,39,47,48^. Hence, the presence of leader cells is likely to depend on more factors than the length scale *λ*. By contrast, $\sqrt {h/\kappa }$ at the monolayer edge, which we studied here, depends on the interactions between many cells. As such, $\sqrt {h/\kappa }$ is largely unaffected by differences in phenotype of any one cell. Importantly, the length scale *λ* also depends on interactions between multiple cells, meaning it is appropriate to use $\sqrt {h/\kappa }$ as an indicator for length scale *λ*.

Results in Fig. 4 indicated that increased substrate stiffness caused decreased $\sqrt {h/\kappa }$, meaning smaller length scale *λ*, at the edge of the cell layer. As *λ* describes the distance over which viscous cell stresses are transmitted, it may seem logical to expect that *λ* would be proportional to the velocity correlation length, in which case increased substrate stiffness would reduce the velocity correlation length. This reasoning contrasts with data in Fig. 2 showing that increased substrate stiffness caused an increase in velocity correlation length. This apparent contradiction is resolved by considering that the change in substrate stiffness has numerous physical effects. In our data, increasing substrate stiffness increased both magnitudes and correlation lengths of traction and tension (Fig. 5). In a recent study, we demonstrated that the traction correlation length has a strong effect on the velocity correlation length^42^, which would explain why the velocity correlation length increased with increasing stiffness in Fig. 2. This explanation, however, brings up a new question, which is why, given the numerous effects of changing substrate stiffness, the data quantifying $\sqrt {h/\kappa }$ at the edge of the cell layer were consistent with interpretation through length scale *λ*. We reason that $\sqrt {h/\kappa }$ at the edge of a cell layer may be robust to changes in the correlation lengths of forces (traction and tension), because the forces at the edge of the cell layer differ systematically from those within the bulk. In particular, tension at the edge of the cell layer is zero^41,49^, and tractions at the edge of the cell layer orient so as to pull the cells into the free space^50^. Importantly, the model by Alert et al.^18^, which relates *λ* to $\sqrt {h/\kappa }$, accounts for these differences in forces at the edge of the cell layer.

A remaining question is how exactly increasing substrate stiffness increased the friction. Considering our data with the expression *Nkvτ*, the increased stiffness seems to have increased both *k* and *N*, but the relative effects of *k* and *N* are not yet clear. Another complication is that the bond lifetime *τ* can be force dependent^51^, in which case the equation *F* = *Nkvτ* would become more complicated, with *τ* depending on *F*, hence producing a more complicated, nonlinear relationship. Sorting out these details would require methods to control the substrate stiffness, bond lifetime, and the number of cell–substrate adhesions independently. Such a method would build on findings of this study, and could lead to an experimental framework to quantify the viscosity and friction, which is an essential step towards relating the collective motion to the forces produced by each cell.

## Methods

### Cell culture

Human keratinocytes (HaCaTs) were provided by Professor Kristyn Masters’ Lab (University of Wisconsin-Madison). For the traction experiments, the HaCaTs stably expressed red fluorescent protein (pHIV-H2BmRFP plasmid, Histone H2B monomeric red fluorescent protein^52^), and were provided by Professor Pamela Kreeger’s Lab (University of Wisconsin-Madison). The cells were maintained in Dulbecco’s modified Eagle’s medium (DMEM, Cellgro 10–013-CV, Corning Inc., Corning, NY, USA) supplemented with 10% fetal bovine serum (FBS, Corning), 100 U/mL penicillin (Corning), and 100 µg/mL streptomycin (Corning) at 37 °C and 5% CO_2_ in an incubator. For all experiments, HaCaTs were used between passages 38 and 48.

### Polyacrylamide substrates

Polyacrylamide substrates were prepared to have a thickness of 150 μm. #1.5 glass bottom dishes (Cellvis, Mountain View, CA, USA) were activated using a 0.3% weight/volume (w/v) concentration 3-(Trimethoxysilyl) propyl methacrylate 98% (Sigma-Aldrich, St. Louis, MO, USA) in 0.2% solution of acetic acid. After 15 min of treatment, the glass bottom dishes were rinsed and left to air dry overnight. To prepare substrates with elastic moduli of approximately 6, 18, and 27 kPa, gel solutions of 10% (w/v) acrylamide (Biorad Laboratories, Hercules, CA, USA) and 0.06%, 0.15%, or 0.3% w/v bisacrylamide (Biorad), respectively, were prepared^53^. Substrates with Young’s moduli of 6 kPa where used for all experiments except where stated otherwise. 20 µL of the respective gel solution was pipetted onto each glass bottom dish for the gel layer. A glass coverslip (18-mm diameter circle) was then placed on top of each gel to ensure the substrate polymerized evenly. Polyacrylamide substrates were stored overnight at 4 °C in deionized water to allow the gels to swell. Once the gels had swelled, the substrates were treated with 50 mg/mL of the covalent cross-linker sulfo-SANPAH (Pierce Biotechnology, Waltham, MA), irradiated with ultraviolet light for 12 min and rinsed with HEPES buffer. Finally, substrates were coated with 500 μL of 0.01 mg/mL type I rat tail collagen (Corning) and incubated at 4 °C overnight prior to cell seeding.

### Magnetic barriers

To constrain the cell monolayers before migration, physical barriers were used (Supplemental Fig. S1). Barriers were either flat or, for Fig. 4a–i, patterned with triangular protrusions of width 40 µm, length 45 µm, and spaced 250–2000 µm apart. The barriers were designed to be magnetic to avoid motion of the barrier during cell seeding and to minimize damage to the cells when the barrier was removed via a magnet placed on top of the dish. To create a barrier that would be magnetic, PDMS (Sylgard 184, Dow Corning, Midland, MI, USA) was mixed with − 200 mesh iron powder at a concentration of 200 mg/mL (Alfa Aesar, Tewksbury, MA, USA) and poured into a plastic petri dish. The PDMS with iron powder was cured for 4 h on a hot plate at 70 °C to make 500–700 µm thick sheets. To ensure that the iron filings did not oxidize, the sheets were spin-coated with an additional thin layer (100 μm) of PDMS on each side. The sheets were then cut into 1 mm wide barriers to be used for the wound healing experiments. The barriers were incubated overnight in 2% Pluronic F-127 (Sigma-Aldrich) to prevent adhesion of the collagen or cells and treated with 70% ethanol prior to use.

The barriers were placed in the center of the collagen I-functionalized polyacrylamide gels and were held in place by a magnet beneath the glass dishes in a custom-designed fixture (Supplemental Fig. S1). Once the barriers were in place, HaCaTs were seeded next to the barrier in a confluent monolayer by pipetting 500 µL of a cell solution (1 × 10^6^ cells/mL) onto each polyacrylamide gel. Cells were then incubated at 37 °C and 5% CO_2_ for 12, 24, or 48 h at which time the barrier was removed. To remove the barrier, the cells were rinsed with cell medium twice, and a second magnet was placed over the center of the dish to lift the barrier off the gel, allowing cell migration into the newly-created space (Supplemental Fig. S1).

For the experiments in Fig. 2, HaCaTs were seeded in a large confluent monolayer onto each polyacrylamide gel. Cells were then incubated at 37 °C and 5% CO_2_ until the start of time lapse imaging. All imaging was conducted in the center of the monolayer, far from all edges.

### Time-lapse microscopy

After barrier removal, wide field phase contrast imaging was performed in a 37 °C 5% CO_2_ humid environment using a custom-built incubator cage. An inverted Eclipse Ti fluorescent microscope run by Nikon NIS-Elements Ar software (Nikon Instruments Inc., Mellville, NY, USA) was used with a 4 × or 10 × objective. Time-lapse phase contrast images were taken every 20 min over a period of 24–72 h to calculate cell velocity and to compute the correlation length of velocity. Phase contrast images were taken at 0 and 24 h of migration to calculate curvature and amplitude of leading edge.

### Curvature, amplitude, and distance between protrusions at the monolayer edge

To compute curvature and amplitude of the monolayer edge, phase contrast images of cell migration were binarized using methods adapted from a prior protocol^54^, enabling the coordinates of the boundary of the cell monolayer to be identified. The coordinates of the cell boundary were used to compute the arc length and the angle over windows of 50 coordinate points. The curvature was computed using linear regression of angle against arc length; the slope was the curvature, *κ*, having units of inverse length, μm^−1^. The RMS of *κ* was computed for each field of view. To compute the amplitude of the monolayer edge, a line was fit to the boundary of the cell monolayer. The distance of the cell boundary to the fitted line, *h*, was computed by an orthogonal projection having units of μm. The RMS of *h* was then computed for each field of view.

The distance between protrusions was measured manually by measuring the length between peaks at the leading edge from the phase contrast images in ImageJ. The average of the distances was computed for each field of view.

### Cell velocities and velocity correlation length

Fast iterative digital image correlation (FIDIC)^55^ was used to compute cell velocity from the time-lapse phase contrast images. The subset size and spacing were either 64 × 64 and 16 pix or 48 × 48 and 12 pix, for images acquired with 10 × and 4 × objectives, respectively. For computing the velocity autocorrelation, for each field of view, the mean of the velocity in that field of view was subtracted from each data point, giving a mean-subtracted velocity, $\vec{v}_{m}$. The normalized spatial autocorrelation of $\vec{v}_{m}$ was computed using the equation

1 $$C\left( r \right) = \frac{{{\Sigma }\left[ {\vec{v}_{{m\left( {\vec{r}^{^{\prime}} } \right)}} \cdot \vec{v}_{{m\left( {\vec{r}^{^{\prime}} + \vec{r}} \right)}} } \right]}}{{{\Sigma }\left[ {\vec{v}_{{m\left( {\vec{r}^{^{\prime}} } \right)}} \cdot \vec{v}_{{m\left( {\vec{r}^{^{\prime}} } \right)}} } \right]}}$$

where $\vec{r}$ and $\vec{r}^{\prime }$ are position vectors with $r = \left| {\vec{r}} \right|$, and symbol ${\Sigma }$ represents a sum over all data points. The correlation length of velocity was determined by the distance at which the velocity correlation dropped to a value of 0.2.

### Cell persistence and directionality

To calculate the persistence of migration, cellular trajectories were computed from cell migration data. The start-to-end distance of the cell trajectory was divided by the traversed path length to give the unitless cell persistence ratio. Here a value of 1 describes persistent, straight-line migration, and a value of 0 describes fully random motion. For cell directionality, the angle of the cellular trajectories was computed, with migration toward the monolayer edge corresponding to an angle of 0°. A histogram of the cell directions was computed for each field of view.

### Immunofluorescent staining and imaging

For experiments with immunofluorescent staining, cells were cultured for different times (12, 24, 48 h), or they were cultured on substrates of different modulus (6, 18, or 27 kPa). At the desired time after barrier removal, the cells were fixed and stained. To fix the cells, HaCaTs were rinsed twice with phosphate-buffered saline (PBS, Corning) and then fixed with 4% paraformaldehyde solution while swirling for 15–20 min. HaCaTs were rinsed twice with PBS for 5–10 min while swirling. After fixation, cells were permeabilized with 0.1% Triton solution.

For staining of focal adhesions, a monoclonal anti-vinculin antibody 1:200 (catalog no. V4505, Sigma-Aldrich) was used. The dishes were incubated overnight at 4 °C, and then an Alexa Fluor 488 1:100 (catalog no. A-11059, Thermo Fisher Scientific, Waltham, MA, USA) secondary antibody was added followed by incubation overnight. For staining of cell–cell adhesions, the monoclonal antibody E-cadherin 24E10 Alexa Fluor 488 conjugate 1:200 (catalog no. 3199S, Cell Signaling Technology) was added and cells were incubated overnight at 4 °C. To stain for actin, 594 Phalloidin Dylight 1:200 (catalog no. 21836, Thermo Fisher Scientific) was added and dishes were incubated for one hour. For all immunofluorescent staining, the antibodies were diluted into a working solution with PBS according to manufacturer instructions.

For immunofluorescent imaging, a Nikon A1R confocal microscope using NIS-Elements Ar software (Nikon) with a 40× numerical aperture 1.15 water immersion objective was used. Image stacks with 0.8 µm step size were taken from the apex to the base of the cells. To check for the presence of an actomyosin cable, actin at the leading edge was imaged. For microscopy of cortical actin and E-cadherin, image stacks were captured through the cell height, and a maximum intensity projection was computed. Images showing focal adhesions are of a single plane near the cell base.

### Quantification of E-cadherin relative intensity

E-cadherin images were analyzed by computing the relative fluorescent intensity of cadherin at the cell–cell junctions to intensity in the cytoplasm of each cell. To define the cell–cell junctions, maximum intensity projections of cortical actin were segmented using Seedwater Segmenter^56^. The segmented cell junctions were dilated by approximately 0.7 μm, enabling the fluorescent intensities in junctional and cytoplasmic regions to be identified. Finally, the relative cadherin intensity was defined as the difference between junctional and cytoplasmic intensity divided by the sum.

### Quantification of normalized vinculin area

Confocal images of vinculin were thresholded to make binary images of vinculin (representative images are shown in Supplemental Fig. S2). The same threshold was used for all images. The ratio of the area of the image containing vinculin to the area of the image containing cells was computed, giving the normalized vinculin area for each image.

### Traction force microscopy and monolayer stress microscopy

Polyacrylamide substrates were prepared for traction force microscopy as described above but with the addition of 0.2 µm fluorescent particles (Dark Red 660/680, Fluospheres, Thermo Fisher Scientific). During gel polymerization, the gels were centrifuged upside down to localize the particles to the top as described in our prior work^57,58^. The traction experiments were performed with the cells expressing red fluorescent protein in their nuclei. Time lapse images were collected by phase contrast microscopy and fluorescent microscopy of both cell nuclei and fluorescent particles. Following the experiment, the cells were released from the substrate using trypsin, and a reference image of the fluorescent particles in the substrate was collected.

FIDIC was used to compute the substrate displacements with respect to the reference image, and tractions were computed using Fourier transform traction cytometry^59^ accounting for the finite substrate thickness^49,60^. Stresses in the plane of the monolayer were computed with monolayer stress microscopy^41,61^, from which we computed the monolayer tension, which was defined as the average of the principal stresses. Prior to computing the RMS of tractions and average monolayer tension, the phase contrast images of the cell layer were segmented to identify the monolayer boundary. RMS tractions and average tension were computed only for areas inside the monolayer boundaries.

To map the tractions to each cell, we used our recently published procedure^42^. Briefly, images of the cell nuclei were segmented using the ImageJ plugin StarDist^62^, and the cell outlines were approximated by creating a Voronoi tessellation based on the centroid of each segmented nucleus. Tractions underneath each Voronoi cell were summed to compute the net traction applied by that cell. The spatial correlation of traction and tension were computed using Eq. (1), but replacing variable $\vec{v}$ with either the unit vector defining the direction of net traction or the scalar tension. Correlation lengths of traction and tension were defined as the distance over which the respective correlations decreased to a value of 0.2.

### Statistical analysis

Statistical comparisons were performed using the non-parametric Kruskal–Wallis test with Bonferroni correction for multiple-comparison. A value of *p* < 0.05 was considered statistically significant. The symbols *, **, and *** are used to denote *p* < 0.05, 0.01, and 0.001, respectively. All data reported are mean ± standard deviation unless otherwise noted.

## Supplementary Information

Supplementary Information.

## Data Availability

The datasets generated are available from the corresponding author on reasonable request.
