# Supplementary material for: Effect of substrate stiffness on friction in collective cell migration
Source: Sci Rep. 2022 Feb 15;12:2474. doi: 10.1038/s41598-022-06504-0 (PMC8847350; doi:10.1038/s41598-022-06504-0)
Supplement: Supplementary file 1 — Supplementary Information. [file 41598_2022_6504_MOESM1_ESM.pdf]

Supplemental Information

Effect of substrate stiffness on friction in collective cell migration

Kelly Vazquez,[1,2] Aashrith Saraswathibhatla,[1] Jacob Notbohm[1,2]

[1]Department of Engineering Physics
[2]Department of Mechanical Engineering
University of Wisconsin–Madison, Madison, WI, USA

Supplemental Figures

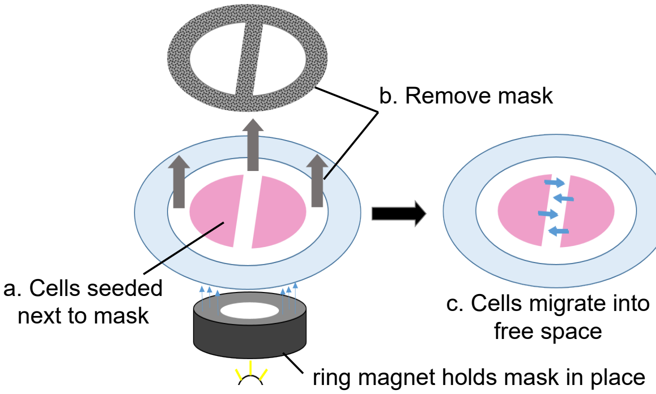

Figure S1: Schematic of experimental setup for experiments with a free edge. (a) HaCaT cells were seeded in confluent monolayers next to PDMS masks embedded with iron filings. The dishes were placed on top of a magnet, which kept the masks in place during cell culture. (b) At the start of each experiment, a second magnet was held above the dish, allowing the mask to lift up from the substrate. (c) After mask removal, cells began to migrate into the free space.

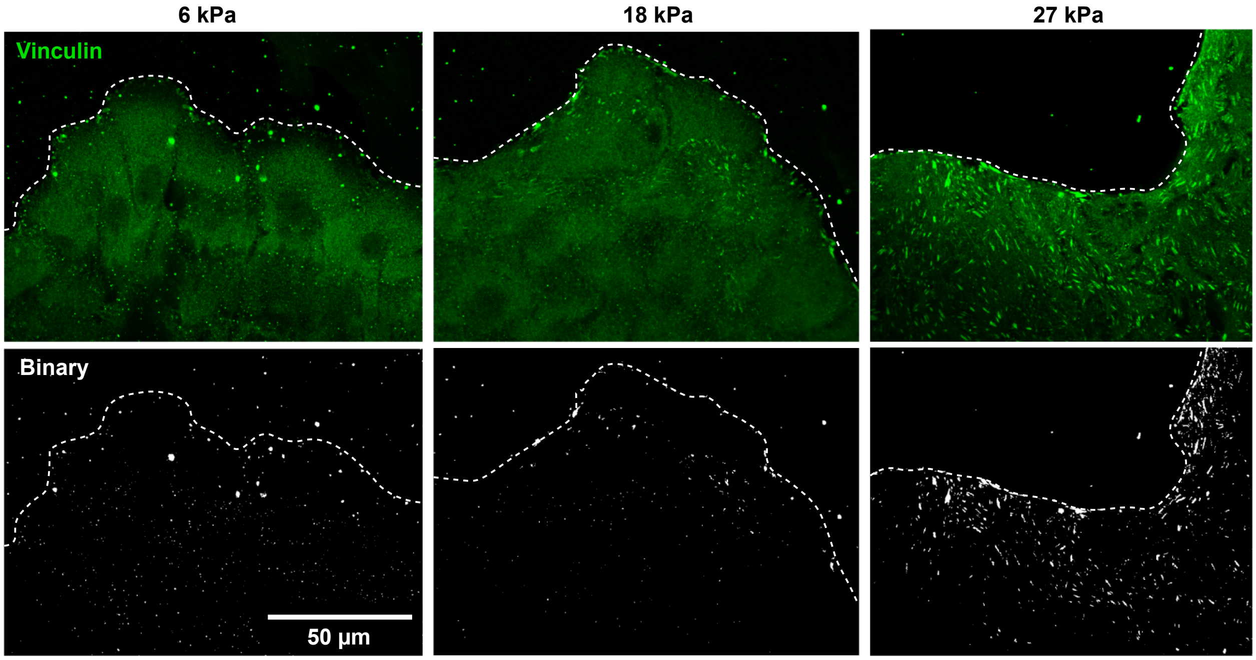

Figure S2. Quantification of focal adhesions for varied substrate stiffness. Images of vinculin (representative images shown in the top row) were thresholded to create binary images (bottom row). The dashed lines represent the edge of the monolayer. Normalized vinculin area was defined as the ratio of the thresholded area within the monolayer to the total monolayer area.

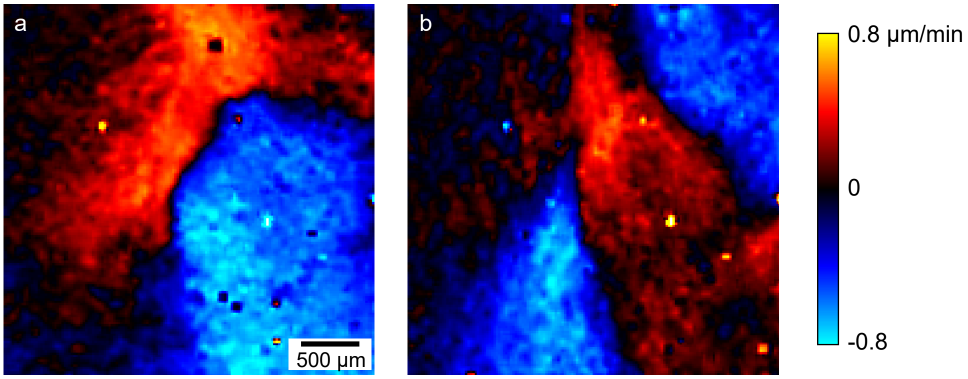

Figure S3. Velocity fields in a large confluent monolayer. Representative color maps of velocity field showing x and y components of velocity (in panels a and b, respectively) for HaCaTs on a substrate of modulus 18 kPa. The different colors (red and blue) indicate regions wherein the cells moved in different directions, and hence define different collectively moving groups of cells.

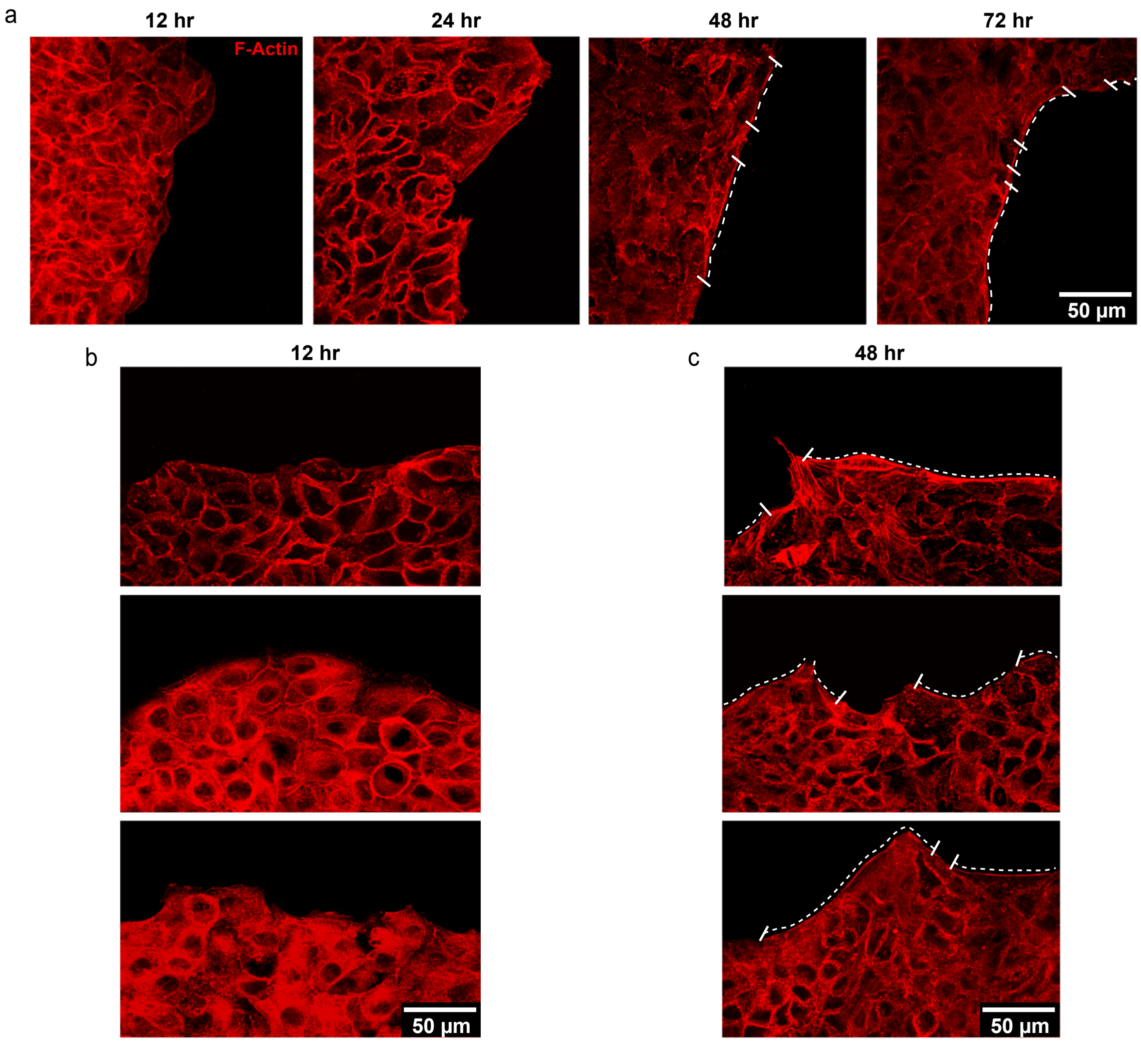

Figure S4. Effect of time in culture on presence of multicellular actin cable. (a) Confocal images of HaCaTs stained for actin immediately after barrier removal. Times indicate the time in culture prior to barrier removal. The white dashed lines in the 48 and 72 hr images indicate the presence of multicellular actin cables with space between the dashed lines indicating breaks in the cables. (b, c) Confocal images of actin after 24 hr of migration for cells that were in culture for (b) 12 or (c) 48 hr prior to barrier removal.

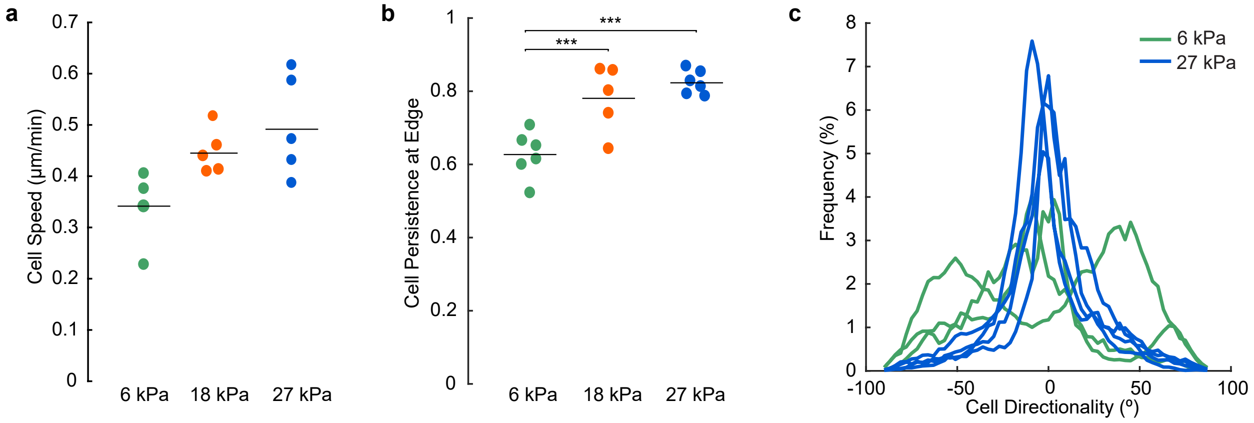

Figure S5. Effect of substrate stiffness on cell speed, persistence, and directionality. (a) Average cell speed for cells on varied substrate stiffness. Each data point represents an average from an independent position. The three groups are not statistically different. (b) Persistence of cells near the free edge of the monolayer on substrates of varying stiffness. A value of 0 indicates random, nonpersistent motion, whereas a value of 1 indicates cells migrating in a straight line. Each data point represents an average from an independent position. (c) Line plots of histograms showing the frequency of cell direction where 0° indicates migration toward the edge of the cell monolayer. Each line represents the distribution for an independent position.

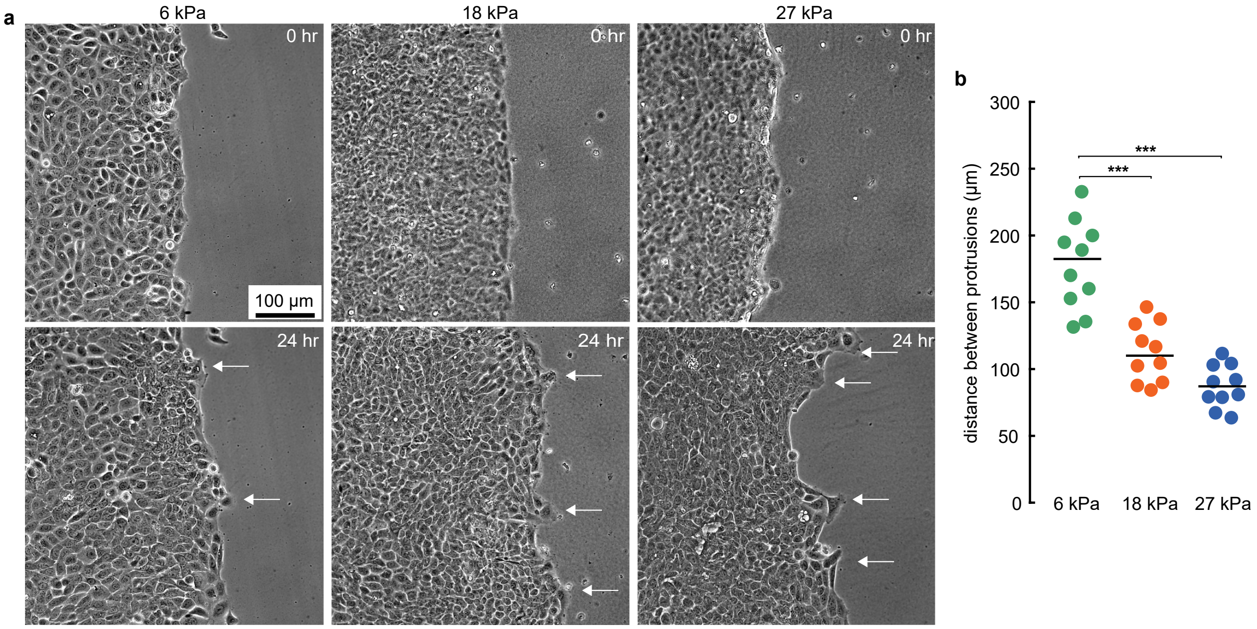

Figure S6. Effect of substrate stiffness on distance between protrusions at edge of cell layer. (a) Representative phase contrast images of HaCaTs on substrates having moduli of 6, 18, and 27 kPa immediately after flat edge barrier removal (top row) and 24 hr after barrier removal (bottom row). White arrows depict visible protrusions at the leading edge. (b) The average distance between protrusions was manually measured for monolayers on substrates of varied stiffness.
